# Diffusion Tensor Imaging of a Median Nerve by Magnetic Resonance: A Pilot Study

**DOI:** 10.3390/life12050748

**Published:** 2022-05-18

**Authors:** Kanza Awais, Žiga Snoj, Erika Cvetko, Igor Serša

**Affiliations:** 1 Jožef Stefan International Postgraduate School, 1000 Ljubljana, Slovenia; kanza628@gmail.com; 2 Department of Radiology, University Medical Centre Ljubljana, 1000 Ljubljana, Slovenia; ziga.snoj@kclj.si; 3 Faculty of Medicine, University of Ljubljana, 1000 Ljubljana, Slovenia; erika.cvetko@mf.uni-lj.si; 4 Jožef Stefan Institute, 1000 Ljubljana, Slovenia

**Keywords:** magnetic resonance microscopy, peripheral nerve anatomy, diffusion tensor imaging, fractional anisotropy, mean diffusivity

## Abstract

The magnetic resonance Diffusion Tensor Imaging (DTI) is a powerful extension of Diffusion Weighted Imaging (DWI) utilizing multiple bipolar gradients, allowing for the evaluation of the microstructural environment of the highly anisotropic tissues. DTI was predominantly used for the assessment of the central nervous system (CNS), but with the advancement in magnetic resonance (MR) hardware and software, it has now become possible to image the peripheral nerves which were difficult to evaluate previously because of their small caliber. This study focuses on the assessment of the human median peripheral nerve ex vivo by DTI microscopy at 9.4 T magnetic field which allowed the evaluation of diffusion eigenvalues, the mean diffusivity and the fractional anisotropy at 35 μm in-plane resolution. The resolution was sufficient for clear depiction of all nerve anatomical structures and therefore further image analysis allowed the obtaining of average values for DT parameters in nerve fascicles (intrafascicular region and perineurium) as well as in the surrounding epineurium. The results confirmed the highest fractional anisotropy of 0.33 and principal diffusion eigenvalue of 1.0 × 10^−9^ m^2^/s in the intrafascicular region, somewhat lower values of 0.27 and 0.95 × 10^−9^ m^2^/s in the perineurium region and close to isotropic with very slow diffusion (0.15 and 0.05 × 10^−9^ m^2^/s) in the epineurium region.

## 1. Introduction

Magnetic Resonance Imaging (MRI) is a well-established imaging modality capable of providing high resolution structural and functional images of the tissues in the human body. This technique is relatively safer than other imaging modalities such as X-rays and CT scanning due to the absence of harmful radiation. Conventionally, pathologies have been evaluated using a combination of medical history, clinical and diagnostic assessment, but advances in MRI have increased the reproducibility of findings, post-surgical evaluation and simultaneous assessment of the bones and soft tissue, thus, the utilization of MRI is increasing with time. One of the important advantages of MRI over other radiological methods is also in the ability to study a wide range of pathologies, which is made possible by the high flexibility in designing pathology-specific imaging methods. One such group are also neurological disorders that can be efficiently studied by Diffusion Weighted Imaging (DWI) and its powerful extension Diffusion Tensor Imaging (DTI).

DWI employs bipolar gradient pulses for the assessment of diffusion, i.e., random motion of molecules caused by temperature. The result of this is signal attenuation in DWI, which is dependent on the diffusion constant of water molecules in the tissue and on the instrumental parameter *b*-value, which incorporates the effects of the bipolar diffusion gradient, specifically its amplitude and duration. Tissues with faster diffusion will have greater signal attenuation and this will also be increased with the higher *b*-values. DWI is, particularly, useful in neuroimaging for the assessment of stroke [1]. The affected region of brain appears brighter in the DWI than the normal part. From at least two different DW images, in the same slice but with different *b*-values, it is also possible to calculate the map of the Apparent Diffusion Coefficient (ADC), which is a quantitative measure for the diffusion in the tissue. Still quantitative, however, a more advanced method is DTI that employs bipolar gradients in many directions enabling it to visualize the microstructural environment of the highly anisotropic tissues [2,3]. DTI is useful in the assessment of pathologies in the white matter tract in the CNS [4], skeletal muscles and the peripheral nerve pathologies such as carpel tunnel syndrome [5,6].

The imaging of acute nerve injuries, compression, inflammatory neuropathies and peripheral nerve tumors have been made easier to evaluate with the advancements in DTI. Skorpil et al. was the first who demonstrated the feasibility of using DTI in the assessment of the sciatic nerve [7]. Hiltunen et al. then subsequently demonstrated the possibility of utilizing DTI in the evaluation of peripheral nerves at the wrist, knee and ankle [8]. Khalil et al. demonstrated the assessment of the median nerve in healthy volunteers and patients with Carpel Tunnel Syndrome (CTS), showing a significant decrease in the fractional anisotropy (FA) value in the patients with CTS but no change in the ADC values [6]. Bulut et al. evaluated FA and ADC values in healthy volunteers and patients with mild, moderate and severe CTS, demonstrating a significant difference in FA and ADC values not only between the patients with CTS and healthy volunteers but also between the patients whose CTS differed in severity [5]. Bäumer et al. has recently demonstrated the utility of DTI in the diagnosis of ulnar nerve entrapment at the cubital tunnel [9]. Yamasaki et al. compared DTI at 7 T with the histology in the rabbit model of the sciatic nerve crush injury [10]. Morisaki et al. demonstrated similar findings at the 4.7 T system in a rat model of sciatic nerve crush injury with a decrease in FA value and an increase in the radial diffusivity following FA value augmentation and a decrease in radial diffusivity in a group with temporary injury [11]. Meek et al. reported a DTI finding utilizing the fiber tractography in a case following median nerve repair for nerve laceration at the wrist to demonstrate progressive distal extension of the nerve over time [12]. Chen et al. was able to identify C5 to C8 nerve roots using tractography [13]. To access lumbar nerve roots, Budzik et al. utilized reduced field of view imaging and obtained a signal from the region of interest (ROI) [14]. Chhabra et al. used DWI and DTI to demonstrate a variety of peripheral nerve tumors [15]. In case of soft tissue tumors in close proximity to the peripheral nerves, Kasprian et al. assessed the utility of DTI in identifying the peripheral nerve infiltration [16]. Kakuda et al. showed a significant decrease in FA values of tibial nerve in subjects with chronic inflammatory demyelinating polyneuropathy compared with the healthy controls [17], thus signifying pathological utility and wide-adaptability of the DTI technique.

## 2. Theory

As follows from the Bloch–Torrey equation for nuclear magnetic resonance (NMR) signal [18], the signal attenuation of the spin–echo is because of the diffusion in the presence of magnetic field gradient equal to

(1) $$A=\gamma^{2}\int_{0}^{TE}{\left(\vec{F}(t^{\prime })-2H(t^{\prime }-TE/2)\vec{f}\right)}^{T}\;\underline{D}\;\left(\vec{F}(t^{\prime })-2H(t^{\prime }-TE/2)\vec{f}\right)dt^{\prime },$$

where *γ* is gyromagnetic ratio of the observed nuclei, *TE*, is time from the signal excitation to the spin–echo, *H*(*t*) is the Heaviside step function

(2) $$H(t)=\{\begin{array}{c} 0\;;\;t<0\\ 1\;;\;t\geq 0 \end{array}$$

and $\underline{D}$ is the second-order diffusion tensor equal to

(3) $$\underline{D}=\left[\begin{array}{ccc} D_{xx} & D_{xy} & D_{xz}\\ D_{yx} & D_{yy} & D_{yz}\\ D_{zx} & D_{zy} & D_{zz} \end{array}\right].$$

Here, vectors $\vec{F}(t^{\prime })$ and $\vec{f}$ are defined as

(4) $$\vec{F}(t^{\prime })=\left[\begin{array}{c} \int_{0}^{t^{\prime }}G_{x}(t^{\prime \prime })dt^{\prime \prime }\\ \int_{0}^{t^{\prime }}G_{y}(t^{\prime \prime })dt^{\prime \prime }\\ \int_{0}^{t^{\prime }}G_{z}(t^{\prime \prime })dt^{\prime \prime } \end{array}\right],\;\;\vec{f}=\vec{F}(TE/2).$$

As proposed in [2], Equation (1) can be significantly simplified by introducing a second-order tensor $\underline{b}$ defined as

(5) $$\underline{b}=\gamma^{2}\int_{0}^{TE}\left(\vec{F}(t^{\prime })-2H(t^{\prime }-TE/2)\vec{f}\right){\left(\vec{F}(t^{\prime })-2H(t^{\prime }-TE/2)\vec{f}\right)}^{T}dt^{\prime }$$

With the introduction of the tensor $\underline{b}$, the signal attenuation in Equation (1) can be expressed by double dot product of tensors (analogous to dot product of vectors) $\underline{b}$ and $\underline{D}$

(6) $$A=\sum_{i,j}b_{ij}D_{ij}=\underline{b}:\underline{D}.$$

Since both tensors are symmetric, they have six independent components, so that this product can be transformed to a standard dot product

(7) $$A=\vec{b}\cdot \vec{D}$$

of six-component vectors defined as

(8) $$\vec{D}=(D_{xx},D_{yy},D_{zz},D_{xy},D_{xz},D_{yz}),\;\vec{b}=(b_{xx},b_{yy},b_{zz},2b_{xy},2b_{xz},2b_{yz}).$$

**Figure 1:**
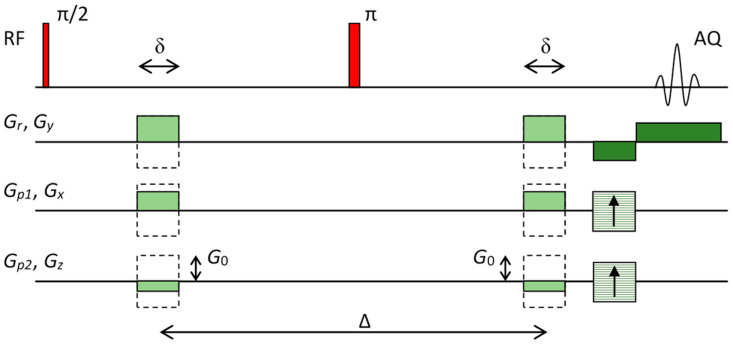
Imaging pulse sequence that was used for DTI. The sequence is based on the standard 3D spin–echo sequence with the addition of diffusion gradients.

Equation (7) is the fundamental equation for calculation of the diffusion tensor (vector $\vec{D}$). Since this tensor has six independent components, at least six signal attenuations $A_{i}$ must be measured with different vectors ${\vec{b}}_{i}$. Individual attenuation is usually measured in a spin–echo experiment (Figure 1) where a pair of diffusion magnetic field gradient pulse is added to the standard spin–echo imaging sequence. Two echo signals are acquired with this sequence: one with the diffusion gradients off and the other with the diffusion gradients on that yield signals $S_{b=0}$ and $S_{i}$, respectively. These two signals can be mathematically expressed by

(9) $$S_{b=0}=S_{0}\exp(-TE/T_{2}),\;S_{i}=S_{0}\exp(-TE/T_{2})\exp(-{\vec{b}}_{i}\cdot \vec{D}).$$

Here $S_{0}$ denotes the signal that follows immediately after the signal excitation, TE is the spin–echo time and $T_{2}$ is the transversal NMR relaxation time. Signal attenuation $A_{i}$ is then obtained by calculating negative natural logarithm of the quotient of these two signals

(10) $$A_{i}=-\ln\left(\frac{S_{i}}{S_{b=0}}\right)={\vec{b}}_{i}\cdot \vec{D}.$$

For the pulse sequence in Figure 1, diffusion gradients have equal amplitudes but different directions. These gradients can be expressed as ${\vec{G}}_{i}=G_{0}\;{\vec{n}}_{i}$ where $G_{0}$ are gradient amplitudes and normal vectors ${\vec{n}}_{i}=(n_{i,x},n_{i,y},n_{i,z})$, $\left|{\vec{n}}_{i}\right|=1$ define gradient directions. For this case, the corresponding vectors ${\vec{b}}_{i}$ can be expressed as

(11) $${\vec{b}}_{i}=b\left(n_{i,x}^{2},n_{i,y}^{2},n_{i,z}^{2},2n_{i,x}n_{i,y},2n_{i,x}n_{i,z},2n_{i,y}n_{i,z}\right),$$

where the *b*-value is equal to

(12) $$b=\gamma^{2}G_{0}{}^{2}\delta^{2}(\Delta -\delta /3).$$

Equation (10) for at least six different attenuations and the corresponding *b*-vectors (i=1…N, N≥6) that must be solved to determine diffusion tensor in general form an overdetermined system

(13) $$\left[\begin{array}{c} A_{1}\\ A_{2}\\ \vdots \\ A_{N} \end{array}\right]=\left[\begin{array}{c} {\vec{b}}_{1}{}^{T}\\ {\vec{b}}_{2}{}^{T}\\ \vdots \\ {\vec{b}}_{N}{}^{T} \end{array}\right]\cdot \vec{D}\;\mathrm{or}\;\vec{A}=\underline{b}\;\vec{D}$$

Here, vectors $\vec{A}$ and $\vec{D}$ have dimensions *N* × 1 and 6 × 1, respectively, while tensor $\underline{b}$ has dimension *N* × 6. The system of equations can be solved by a standard approach for solving over-determined systems of linear equations, i.e., both sides of Equation (13) are multiplied by ${\underline{b}}^{T}$ thus obtaining a quadratic matrix ${\underline{b}}^{T}\underline{b}$ on the right side of the equation. This quadratic matrix can be inverted so that finally vector $\vec{D}$ that holds all components of the diffusion tensor can be expressed as

(14) $$\vec{D}={\left({\underline{b}}^{T}\underline{b}\right)}^{-1}{\underline{b}}^{T}\vec{I}.$$

In the next step of the analysis, the diffusion tensor is diagonalized. As this tensor is symmetric its diagonalization can be carried out with a unitary transition matrix in which columns are eigenvectors of the diffusion tensor

(15) $$\underline{D}\left[\begin{array}{ccc} {\vec{e}}_{1} & {\vec{e}}_{2} & {\vec{e}}_{3} \end{array}\right]=\left[\begin{array}{ccc} D_{1} & 0 & 0\\ 0 & D_{2} & 0\\ 0 & 0 & D_{3} \end{array}\right]\left[\begin{array}{ccc} {\vec{e}}_{1} & {\vec{e}}_{2} & {\vec{e}}_{3} \end{array}\right].$$

The diagonalization of the diffusion tensor does not change its trace; i.e., trace of the tensor is the same in the eigensystem and in the laboratory system

(16) $$D_{xx}+D_{yy}+D_{zz}=Tr(\underline{D})=Tr\left(\left[\begin{array}{ccc} D_{1} & 0 & 0\\ 0 & D_{2} & 0\\ 0 & 0 & D_{3} \end{array}\right]\right)=D_{1}+D_{2}+D_{3}.$$

Mean diffusivity can be defined for the diffusion tensor which can be considered as a measure for an equivalent isotropic diffusion. Mean diffusivity MD is equal to one third of the diffusion tensor trace

(17) $$\mathrm{MD}=\frac{D_{xx}+D_{yy}+D_{zz}}{3}=\frac{D_{1}+D_{2}+D_{3}}{3}.$$

In case of isotropic diffusion, mean diffusivity is equal to $\mathrm{MD}=D_{1}=D_{2}=D_{3}$. If diffusion is anisotropic, the degree of its fractional anisotropy can be determined by expression

(18) $$\mathrm{FA}=\sqrt{\frac{3}{2}\frac{{\left(D_{1}-\mathrm{MD}\right)}^{2}+{\left(D_{2}-\mathrm{MD}\right)}^{2}+{\left(D_{3}-\mathrm{MD}\right)}^{2}}{D_{1}{}^{2}+D_{2}{}^{2}+D_{3}{}^{2}}}$$

The value of FA is between 0 and 1; FA = 0 corresponds to complete isotropic diffusion while FA = 1 corresponds to the most anisotropic diffusion.

## 3. Materials and Methods

### 3.1. Nerve Samples

This pilot study was performed on a single human median nerve that was obtained from the distal part of the upper arm of a body donor (12 h after death) at the Institute of Anatomy, Faculty of Medicine, University of Ljubljana. After drying on the filter paper, the nerve sample was fast frozen using the standard procedure which includes immersion of the nerve in liquid nitrogen and is followed immediately by its storage at −80 °C. Immediately prior to the experiment, an approximately 1 cm long section of the nerve was thawed to room temperature of 22 °C and then inserted in an NMR glass tube with a diameter of 1 cm. To prevent degradation of the sample during MRI scanning, the tube containing the median nerve was filled with fluorinated fluid (Galden SV90, Solvay, Brussels, Belgium). This fluid does not produce any NMR signal and is, therefore, ideal for this purpose. As the fluid is denser than biological tissues, fixation of the nerve with plastic inserts was needed to compensate buoyancy. The tube with the nerve sample was inserted in the 1 cm NMR probe and then placed in the NMR magnet. This study was approved by National Medical Ethics Committee (approval no. 0120-239/2020/3) at the Ministry of Health, Republic of Slovenia.

### 3.2. Diffusion Tensor Imaging

DTI was performed on a system for magnetic resonance microscopy that consisted of a 400 MHz wide-bore vertical superconducting magnet (Jastec Superconductor Technology, Kobe, Japan), Micro 2.5 probe for MR microscopy (Bruker, Ettlingen, Germany) and Redstone NMR/MRI spectrometer (Tecmag, Houston TX, USA). DTI scanning was performed with a pulse sequence based on a three-dimensional spin–echo imaging sequence with added diffusion gradients (Figure 1). Parameters of the sequence were: TE/TR = 36/880 ms, *δ* = 3 ms, Δ = 27 ms, *G*_0_ = 0.26 T/m, *b* = 1150 s/mm^2^ in 19 different gradient directions and one image with *b* = 0, field of view (FOV): 9 × 4.5 × 10 mm^3^, imaging matrix 256 × 128 × 16, signal averages: 4, scan time: 1 day and 16 h. Image resolution was equal to 35 μm along the in-plane directions and the slice thickness was equal to 625 μm. Directions of 19 different diffusion gradients are shown in Table 1; all diffusion gradients had identical amplitudes of *G*_0_ and therefore the same *b*-value.

### 3.3. DTI Calculation and Image Processing

DTI maps of the diffusion eigen values *D*_1_, *D*_2_, *D*_3_, their corresponding eigen vectors, mean diffusivity MD and the fractional anisotropy FA were calculated by C-code software written specifically for this study by the authors. In this software, the solution of Equations (14)–(18) was implemented using the numerical methods [19]. The calculated maps were analyzed for mean values and their errors by ImageJ digital image processing program (NIH, Bethesda, MD, USA). The illustration of DTI data by diffusion ellipsoids was carried out by utilizing the ray-tracing POV-Ray software (open source) for creating three-dimensional (3D) graphics. More details about this software along with its code are included in the Supplementary Materials.

## 4. Results

**Figure 2:**
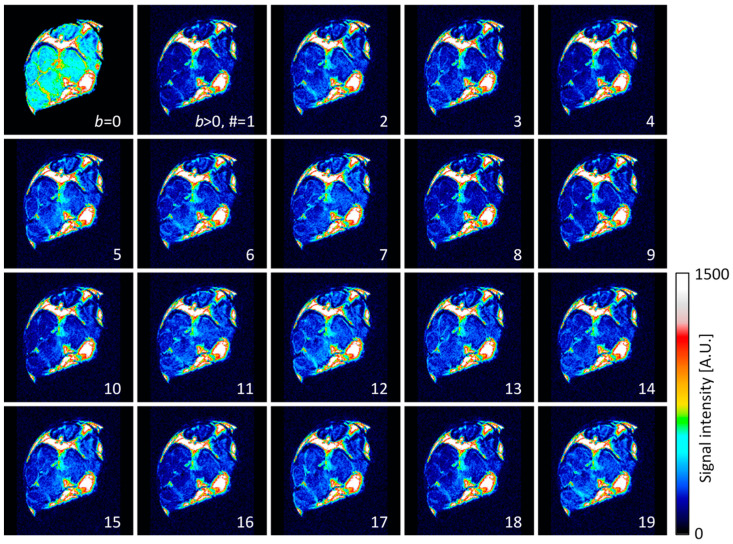
Diffusion-weighted images of the median nerve that correspond to the diffusion gradients in Table 1. Images were acquired with in-plane resolution of 35 μm in central transversal 0.6 mm thick slice across the nerve. The diffusion-weighting parameter *b* was equal to 1150 s/mm^2^.

The diffusion-weighted images of the central transversal slice across the nerve sample are shown in Figure 2. All the images, except for the first one in the top left corner, were acquired with the same diffusion weight of *b* = 1150 s/mm^2^, however, with different diffusion gradient directions that are given in Table 1. The first image has no diffusion weight (*b* = 0) and has on average more than double the signal compared to the images with diffusion weight. The signal reduction is especially apparent in the intrafascicular region, while it is practically negligible in the epineurium region. Higher signal reduction coincides with the regions of faster diffusion (Table 2).

**Figure 3:**
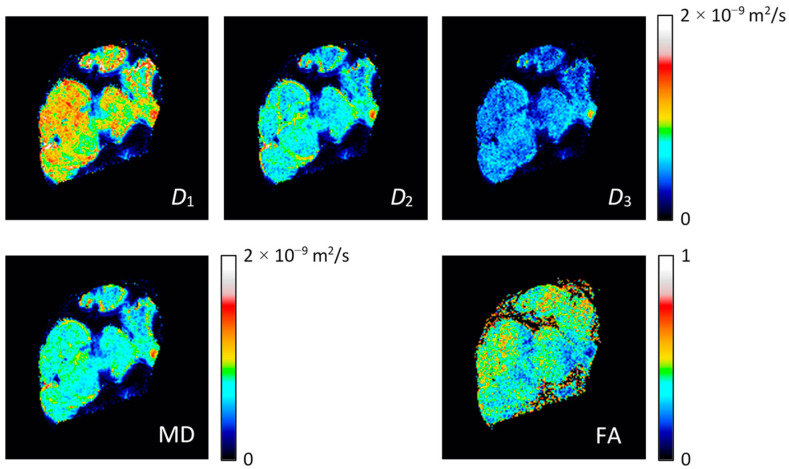
Eigen values of diffusion tensor *D*_1_, *D*_2_ and *D*_3_ (first row) and mean diffusivity MD and fractional anisotropy FA (second row) of the median nerve. The maps were calculated from the DWI data in Figure 2.

The images in Figure 2 were used as an input for the calculation of the diffusion tensor and its diagonalization using Equations (14)–(18). Results for these calculations are shown in Figure 3 with the images of diffusion tensor eigenvalues *D*_1_, *D*_2_, *D*_3_, mean diffusivity MD and fractional anisotropy FA. From these images, it can be inferred that the first eigenvalue *D*_1_ is on average considerably larger than the second and the third eigenvalue, *D*_2_ and *D*_3_. These two eigenvalues are also closer in values to each other. Eigenvalue *D*_1_ is the largest in the intrafascicular region, which has also the highest FA. Lower values of *D*_1_ and also of FA were found in the perineurium region. The values of eigenvalues *D*_2_ and *D*_3_ were found to be higher in the perineurium than in the intrafascicular region. Diffusion values (of all eigenvalues) were found to be the lowest and practically below the detection threshold in the epineurium region.

**Figure 4:**
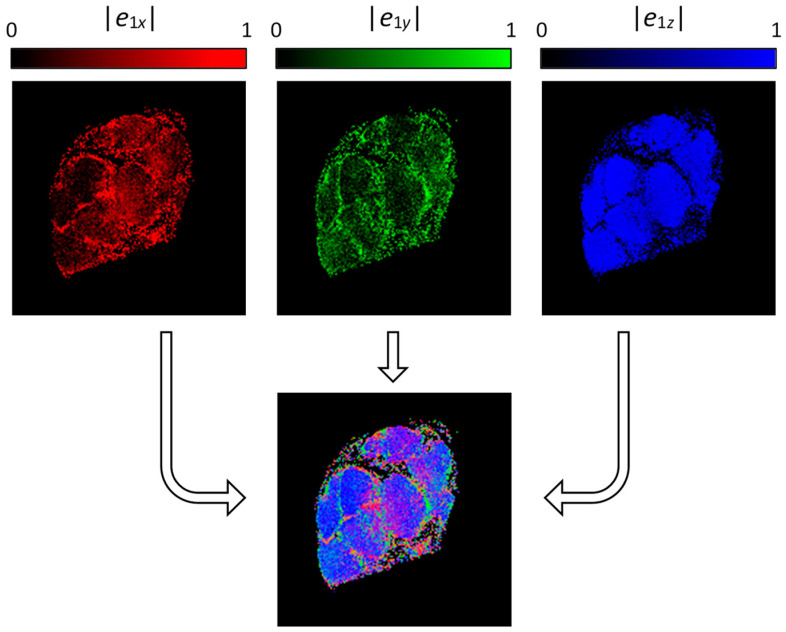
Components of the first eigenvector shown by absolute values for *e*_1*x*_ (red), *e*_1*y*_ (green) and *e*_1*z*_ (blue). The maps of components were used to calculate a composite (RGB) image where pixel color indicates the eigenvector orientation.

Results for the eigenvalue calculation are also eigenvectors ${\vec{e}}_{1},\;{\vec{e}}_{2},\;{\vec{e}}_{3}$, which are mutually perpendicular unit vectors and their directions correspond to the diffusion rates of the eigenvalues *D*_1_, *D*_2_, *D*_3_, respectively. The first eigenvector ${\vec{e}}_{1}$ has direction of the fastest diffusion, i.e., of eigenvalue *D*_1_. In Figure 4, the top row scale defines the *x*, *y* and *z* components of the first eigenvector ${\vec{e}}_{1}$ by red-, green- and blue-scaled images, while in the bottom segment illustrates the composite RBG image of all three previous images. In this composite image, the blue color dominates, especially in the intrafascicular region. This indicates that the diffusion in these regions is the fastest along the *z*-direction, i.e., along nerve fibers, which is an expected result.

**Figure 5:**
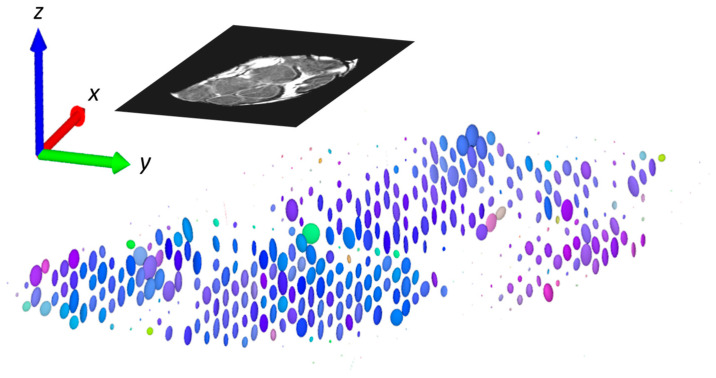
Diffusion tensor in pixels of selected slice represented by ellipsoids. Axes of ellipsoids are proportional to diffusion eigenvalues, while direction of the axes (orientations of the ellipsoids) corresponds to the directions of eigenvectors. Color of the ellipsoids is determined by the composite RGB color as described in Figure 4.

Another presentation, which combines all the existing information, is demonstration of the diffusion tensor by diffusion ellipsoids as shown in Figure 5. The values and orientations for the ellipsoid axes match the eigenvalues and eigenvectors, while their color scheme is orientation-dependent and is determined by the same principle as for the composite RGB color specified in Figure 4. Again, it can be seen that the diffusion is the fastest and predominately oriented along the *z*-direction (along the nerve fibers) in the intrafascicular region, while in the other regions (perineurium), the directionality (anisotropy) of diffusion is not so high. The slow rate of diffusion in the epineurium region can be observed in this image by exceptionally small ellipsoids that appear like small dots.

**Figure 6:**
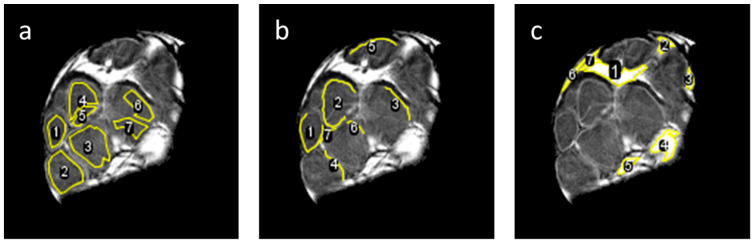
Selected regions of interest (ROIs) in intrafascicular (**a**), perineurium (**b**) and epineurium (**c**) anatomical regions of the median nerve in which the DTI parameters shown in Table 2 were calculated. Note that ROIs for the perineurium regions are only curved lines, one pixel (35 μm) thick.

More precise quantitative evaluation of the results was carried out by the region of interest (ROI) analysis of DWI signals from the intrafascicular, perineurium and epineurium regions. This was conducted in seven different ROIs for each of these three anatomical regions (Figure 6) by calculation of diffusion eigenvalues *D*_1_, *D*_2_, *D*_3_, mean diffusivity MD and fractional anisotropy FA from the corresponding DWI signals shown in Figure 2. The obtained results are presented in terms of means and standard deviations in Table 2. From these results, it can be seen that in the intrafascicular region, *D*_1_ value of 1.00 × 10^−9^ m^2^/s was 67% higher than *D*_2_ and 85% higher than *D*_3_. The intrafascicular region has also the highest FA. An almost identical eigenvalue *D*_1_, however, only 22% higher than *D*_2_ and 85% higher than *D*_3_, was obtained for the perineurium region. The perineurium region has therefore slightly lower FA than the intrafascicular region, while the MDs of both regions are quite similar 0.71 × 10^−9^ m^2^/s vs. 0.77 × 10^−9^ m^2^/s (intrafascicular region vs. perineurium). The ROI analysis verified that the epineurium region has the slowest diffusion of all three analyzed regions. Its MD of 0.05 × 10^−9^ m^2^/s is approximately 15-times lower than the MDs of the intrafascicular and epineurium regions. FA of the epineurium region of 0.15 is also the lowest of all three regions. This value is close to the isotropic diffusion considering the influence of noise in the measured FA.

## 5. Discussion

In this study, DTI microscopy at 9.4 T was performed on the human median nerve ex vivo. Use of high magnetic field enabled high spatial resolution up to 35 μm and therefore a clear visualization of all the major peripheral nerve anatomical structures were apparent, not only in conventional proton-density and DW images, but also in the DT images. Finally, the DTI parameters, such as diffusion eigenvalues *D*_1_, *D*_2_, *D*_3_, mean diffusivity (MD) and the fractional anisotropy (FA), for the three different anatomical regions of the nerve, i.e., intrafascicular, perineurium and epineurium regions, were extracted. Since this was a pilot study, all the research was concluded on an individual median nerve sample from a single donor. Thus, the results obtained are mainly orientational and therefore do not include the possible intra- and inter-subject differences.

As the peripheral nerves are small structures, the conventional clinical MRI scanners can only provide resolution-limited results that merely allow approximate visualization and analysis of the peripheral nerve anatomical structures, e.g., fascicles [20]. Much better results considering the spatial resolution can be obtained by MR microscopy [21], but only at the cost of limiting the extracted nerve samples and thus to ex vivo studies. While MR microscopy can provide anatomical images of spatial resolutions that approach those of optical microscopy, DTI is more challenging as it is very sensitive to noise and requires longer scan times due to scanning in many different diffusion gradient directions. The DW images of poor signal to noise ratio (SNR) or insufficient different diffusion gradient directions (high condition numbers) may lead to overestimated diffusion values and an increased FA [22]. A problem of low SNR is often encountered in MR microscopy. It can be mitigated in hardware by using higher field magnets or more advanced detection systems (cryoprobes), while simpler solutions rely upon modifying the scan parameters, e.g., signal averaging (time consuming) or simply image resolution reduction. In the present experiment, in-plane resolution was 35 μm and the slice thickness was 0.6 mm, which yielded SNR in DW images of 11.5 and 3.7 for the intrafascicular region with *b* = 0 and *b* = 1150 s/mm^2^, respectively. However, DW signals of the regions in Figure 6 had, for the square root of the number of pixels in the region, higher SNRs, e.g., on average 16-times higher for the intrafascicular region. The DTI parameters obtained by calculating the DW signals from the regions (Table 2) are therefore different and more accurate than the corresponding values obtained as region averages of DTI maps (Figure 3). The median nerve is the biggest nerve of the upper extremity, while radial and ulnar nerves are smaller in comparison to it in the upper arm, both nerves could be depicted with similar parameters as described above [23]. However, branches of these nerves are considerably smaller and would therefore require imaging with even higher spatial resolution, which would inevitably lead to the reduction in SNR for DW images and thus render DTI less accurate, or on the contrary, longer scan times due to more signal averaging to maintain the accuracy of DTI.

A long scan time of 1 day and 16 h raised some other problems. One of them was a need for assuring the sample stability during scanning. Small biological samples can severely desiccate and consequently shrink. To prevent this, they can be wrapped in a water impermeable (nonmetallic) foil or be better immersed in liquid. For most other imaging techniques, immersion in physiological solution or in formaldehyde would be optimal. However, for MRI, this is not the case as these liquids contain hydrogen atoms and therefore produce a significant signal that can be comparable or higher to that of the sample. This results in non-optimal receiver gain settings and the obtained images would have poor contrast between the sample and the surrounding liquid. To avoid these problems, a special perfluorinated liquid (Galden SV90) was used that has all hydrogen atoms replaced by fluorine atoms and therefore does not produce any MR detectable signal. This liquid also reduces the magnetic susceptibility problems that usually appear on the sample surfaces.

The use of perfluorinated liquid greatly reduced the sample’s stability problems; however, some minor sample shrinkage of the order of one pixel during sample scanning still remained. This issue was tackled by using an innovative scanning strategy, namely, instead of scanning all 20 images (in different diffusion gradient directions and one with *b* = 0) sequentially, they were all scanned simultaneously. This was achieved by reordering the scanning loops; the inner loop was for the directions of diffusion gradient; the middle loop was for the first phase encoding gradient; and the outer loop was for the second phase encoding gradient. In case of sample shrinkage, part of image resolution would be lost; however, this loss would be the same in all the DW images and therefore would not significantly affect the DTI calculation.

Another difficulty in this study refers to that instead of body temperature, the nerve sample was scanned at a room temperature of 22 °C. As diffusion is temperature dependent and faster at higher temperature, the obtained diffusion values are lower than those expected at the body temperature. A rough estimate for how much the diffusion at the body temperature is faster than at the room temperature provide measurements of diffusion temperature dependency in water, where the increase in diffusion between these two temperatures is equal to 40% [24].

As previously discussed, this study was performed on a frozen median nerve sample that was thawed to room temperature prior to its preparation for MR scanning. A better alternative (or at least an argument) to this would be the use of fresh nerves. Our preliminary results from a similar study on fresh median nerves had shown differences in DTI, especially in the FA parameter which was significantly higher than that observed for the frozen median nerve in the concurrent study. This difference can be explained by the cell structure damage due to the fast-freezing/thawing cycle, which might result in cell rupture, and therefore an increased extra- to intra-cellular ratio contributing towards the reduction in FA.

This study was performed ex vivo and at room temperature. Its adaptation to in vivo conditions is associated with several challenges that are currently unsolved. For example, the currently used clinical MRI scanners do not provide sufficient resolution for a quality depiction of small structures such as nerve fascicles, especially not in the clinically acceptable scan time. Another challenge is to provide quality zoomed images inside a human body. The gradients in the clinical scanners are still considerably weaker than those used in the present study. In the in vivo diffusion experiments, the pulse sequence optimization for the reduction in possible motion artifacts is also a considerable challenge.

## 6. Conclusions

This study demonstrated that DTI of a peripheral nerve ex vivo is feasible with the use of the current state of the art equipment for MR microscopy. With the anticipated future development of the clinical MRI hardware, it is expected that similar studies could also be performed in vivo in future. However, this is conditioned with several open challenges in the development of the new more advanced MRI hardware that would allow high spatial resolution imaging of smaller structures in the human body at acceptable scan times.

## Figures and Tables

**Table 1:** List of 19 different diffusion magnetic field gradients that were used for DTI. All the gradients have identical amplitudes of *G*_0_ but different directions.

Number	*G_x_*/*G*_0_	*G_y_*/*G*_0_	*G_z_*/*G*_0_
1	−0.0620	0.2380	0.9693
2	0.6092	0.7125	0.3483
3	−0.7878	0.3181	−0.5274
4	0.0636	0.1273	0.9898
5	−0.8853	−0.0582	0.4613
6	−0.1428	−0.8146	0.5622
7	0.4337	−0.8094	−0.3959
8	0.2289	−0.4735	0.8505
9	−0.6445	−0.2860	−0.7091
10	−0.4461	0.3051	0.8414
11	0.0199	−0.9966	−0.0799
12	−0.7535	−0.5805	0.3086
13	−0.8716	0.4899	−0.0185
14	−0.4636	0.7949	−0.3914
15	−0.7682	0.4252	0.4787
16	−0.1220	−0.6652	−0.7367
17	−0.9632	−0.1645	−0.2125
18	0.3718	0.3251	−0.8696
19	−0.4298	−0.8959	0.1125

**Table 2:** DTI parameters and their average for different ROIs in intrafascicular, perineurium and epineurium anatomical regions of the median nerve.

Intrafascicular region							
Number	Area [mm^2^]	Signal [A.U.]	*D*_1_	*D*_2_	*D*_3_	MD	FA [0–1]
			[10^−9^ m^2^/s]				
1	0.22	420	1.07	0.60	0.51	0.73	0.39
2	0.53	477	1.00	0.63	0.59	0.74	0.29
3	0.60	511	0.92	0.64	0.56	0.71	0.27
4	0.31	433	1.05	0.57	0.50	0.71	0.40
5	0.11	472	1.05	0.59	0.55	0.73	0.36
6	0.23	473	0.92	0.62	0.56	0.70	0.27
7	0.19	494	0.96	0.58	0.53	0.69	0.32
		469 ± 32	1.00 ± 0.06	0.60 ± 0.02	0.54 ± 0.03	0.71 ± 0.02	0.33 ± 0.06
Perineurium region							
Number	Area [mm^2^]	Signal [A.U.]	*D*_1_	*D*_2_	*D*_3_	MD	FA [0–1]
			[10^−9^ m^2^/s]				
1	0.06	641	0.92	0.67	0.43	0.68	0.35
2	0.08	672	1.09	0.76	0.70	0.85	0.25
3	0.04	708	1.04	0.96	0.56	0.85	0.29
4	0.03	795	0.94	0.87	0.53	0.78	0.27
5	0.04	625	0.84	0.67	0.46	0.66	0.29
6	0.02	676	0.95	0.76	0.60	0.77	0.23
7	0.02	692	1.05	0.93	0.46	0.81	0.36
		687 ± 56	0.98 ± 0.09	0.80 ± 0.11	0.53 ± 0.09	0.77 ± 0.08	0.29 ± 0.05
Epineurium region							
Number	Area [mm^2^]	Signal [A.U.]	*D*_1_	*D*_2_	*D*_3_	MD	FA [0–1]
			[10^−9^ m^2^/s]				
1	0.32	1564	0.09	0.08	0.06	0.08	0.19
2	0.07	1442	0.04	0.03	0.03	0.03	0.20
3	0.06	1308	0.07	0.06	0.05	0.06	0.15
4	0.23	1551	0.04	0.03	0.03	0.03	0.12
5	0.07	1495	0.05	0.05	0.04	0.05	0.09
6	0.04	1383	0.04	0.03	0.03	0.03	0.20
7	0.05	1338	0.05	0.04	0.04	0.04	0.13
		1440 ± 101	0.05 ± 0.02	0.05 ± 0.02	0.04 ± 0.01	0.05 ± 0.02	0.15 ± 0.04

## Data Availability

The data presented in this study are available on request from the corresponding author.

## Supplementary Materials

The following supporting information can be downloaded at: https://www.mdpi.com/article/10.3390/life12050748/s1, programs DTI_3D.c (page 3–7), DTI_3D_ellips.c (page 8–12), nerve_ellips.pov (page 13).
