# Supplementary material for: Diffusion Tensor Imaging of a Median Nerve by Magnetic Resonance: A Pilot Study
Source: Life (Basel). 2022 May 18;12(5):748. doi: 10.3390/life12050748 (PMC9143877; doi:10.3390/life12050748)
Supplement: Supplementary file 1 [file life-12-00748-s001.zip › life-1720671-supplementary.pdf]

Supplementary Materials

Diffusion tensor imaging of a median nerve by magnetic resonance: a pilot study

Kanza Awais[1], Žiga Snoj[2], Erika Cvetko[3], Igor Serša[4,3,*]

[1]*Jožef Stefan International Postgraduate School, Ljubljana, Slovenia*

[2]*Department of Radiology, University Medical Centre Ljubljana, Slovenia*

[3]*Faculty of Medicine, University of Ljubljana, Slovenia*

[4]*Jožef Stefan Institute, Ljubljana, Slovenia*

This file contains three computer programs. This first two:

`DTI_3D.c` (pages 3-7)

`DTI_3D_ellips.c` (pages 8-12)

are written in the C programming language. First, the C-code needs compiling to the .exe (executable) version of the program. Once this is done both programs can be run with a command line, e.g.

```
DTI_3D 3D_DWI_12s.raw 19 1146.4 0 0
```

Where the first parameter (`3D_DWI_12s.raw`) is the name of the raw file that contains all DWI images of all slices and for all *b*-vectors. They ordered by b-vectors in the outer loop and then by slices in the inner loop. The raw file should not have any header and should be of a 32-bit Real type. For these programs, raw files were created by the ImageJ program. The second parameter (`19`) corresponds to different gradient directions; in our case this was 19. The third parameter (`1146.4`) is the magnitude of all *b*-vectors (also known as the *b*-value) and it must be given in units of s/mm^2. The last two parameters (here `0 0`) are thresholds for the signal and for the diffusion value, respectively. Voxels with values below any of the thresholds will not be displayed. The signal threshold refers to the image without any diffusion weight (*b*=0), while the diffusion threshold is for selection of voxels where the calculated diffusion values can be trusted (they are nonnegative and above the noise threshold). Parameters that should be used for the DTI_3D_ellips program are identical to these presented above. The third program:

`nerve_ellips.pov` (page 13)

is a POV-Ray program and shows an example how to integrate code calculated by the DTI_3D_ellips program into POV-Ray.

Some parameters, e.g., sizes of image matrices, are given to the c programs as constants and these should be adjusted to correct values prior to the compilation of the program. Both c programs use also six different libraries for numerical calculation that are part of Numerical recipes in C (Press, W.H. *Numerical recipes in C : the art of scientific computing*, 2nd ed.; Cambridge University Press: Cambridge Cambridgeshire ; New York, 1992; pp. xxvi, 994 p).

```
#include <stdio.h>                  // program DTI_3D.c

#include <stdlib.h>
#include <math.h>

#include "../../recipes/nrutil.c"   // libraries of Numerical recipes in C
#include "../../recipes/gaussj.c"
#include "../../recipes/jacobi.c"
#include "../../recipes/eigsrt.c"
#include "../../recipes/svdcmp.c"
#include "../../recipes/piksrt.c"

#define M1 160        // image matrix size is M1 * M2 * M3 (x y z)
#define M2 160
#define M3 12
int N ;               // number of different gradient orientations
#define L 5           // images (data) per pixel

#define sqr(x) ( (x) * (x) )
#define mag( x , y ) sqrtf( (x) * (x) + (y) * (y) )

const float g6[6][3] = { {  0.0000,  0.7071,  0.7071 },   // diffusion gradient directions
                         {  0.0000, -0.7071,  0.7071 },
                         { -0.7071,  0.0000,  0.7071 },
                         { -0.7071,  0.0000, -0.7071 },
                         { -0.7071,  0.7071,  0.0000 },
                         { -0.7071, -0.7071,  0.0000 } } ;

const float g19[19][3] = { { -0.0620,  0.2380,  0.9693 }, // diffusion gradient directions
                           {  0.6092,  0.7125,  0.3483 },
                           { -0.7878,  0.3181, -0.5274 },
                           {  0.0636,  0.1273,  0.9898 },
                           { -0.8853, -0.0582,  0.4613 },
                           { -0.1428, -0.8146,  0.5622 },
                           {  0.4337, -0.8094, -0.3959 },
                           {  0.2289, -0.4735,  0.8505 },
                           { -0.6445, -0.2860, -0.7091 },
                           { -0.4461,  0.3051,  0.8414 },
                           {  0.0199, -0.9966, -0.0799 },
                           { -0.7535, -0.5805,  0.3086 },
                           { -0.8716,  0.4899, -0.0185 },
                           { -0.4636,  0.7949, -0.3914 },
                           { -0.7682,  0.4252,  0.4787 },
                           { -0.1220, -0.6652, -0.7367 },
                           { -0.9632, -0.1645, -0.2125 },
                           {  0.3718,  0.3251, -0.8696 },
                           { -0.4298, -0.8959,  0.1125 } } ;

float swap(unsigned char *src)      // convert big-endian to little-endian
{
       float a;
       unsigned char *dst = (unsigned char *)&a;

       dst[0] = src[3];
       dst[1] = src[2];
       dst[2] = src[1];
       dst[3] = src[0];

       return a;
}

float** define_btbibt( float b0 )   // matrix product on righthand side of Eq. 14
{
       float **b,**bt,**btb ;
       float sum ;
       int i,j,k ;
```

```
	b = matrix( 1, N, 1, 6 ) ;
	bt = matrix( 1, 6, 1, N ) ;
	btb = matrix( 1, 6, 1, 6 ) ;

	switch( N ) {
		case 6 :
		for( i = 0 ; i < N ; i++ ) {
			b[i+1][1] = b0 * g6[i][0] * g6[i][0] ;
			b[i+1][2] = b0 * g6[i][1] * g6[i][1] ;
			b[i+1][3] = b0 * g6[i][2] * g6[i][2] ;
			b[i+1][4] = b0 * 2.0 * g6[i][0] * g6[i][1] ;
			b[i+1][5] = b0 * 2.0 * g6[i][0] * g6[i][2] ;
			b[i+1][6] = b0 * 2.0 * g6[i][1] * g6[i][2] ;
		} break ;
		case 19 :
		for( i = 0 ; i < N ; i++ ) {
			b[i+1][1] = b0 * g19[i][0] * g19[i][0] ;
			b[i+1][2] = b0 * g19[i][1] * g19[i][1] ;
			b[i+1][3] = b0 * g19[i][2] * g19[i][2] ;
			b[i+1][4] = b0 * 2.0 * g19[i][0] * g19[i][1] ;
			b[i+1][5] = b0 * 2.0 * g19[i][0] * g19[i][2] ;
			b[i+1][6] = b0 * 2.0 * g19[i][1] * g19[i][2] ;
		} break ;
		default :
			printf( "No such gradient table!\n" ) ;
			exit( 1 ) ;
	}

	for( i = 1 ; i <= N ; i++ ) {
		for( j = 1 ; j <= 6 ; j++ ) {
			bt[j][i] = b[i][j] ;
		}
	}

	for( i = 1 ; i <= 6 ; i++ ) {
		for( j = 1 ; j <= 6 ; j++ ) {
			sum = 0.0 ;
			for( k = 1 ; k <= N ; k++ ) sum += bt[i][k] * b[k][j] ;
			btb[i][j] = sum ;
		}
	}

	gaussj( btb, 6, bt, N ) ;	// Gauss-Jordan elimination to compute (btb)-1bt
					// the result is stored in bt, btb holds its inverse
	free_matrix( b, 1, N, 1, 6 ) ;
	free_matrix( btb, 1, 6, 1, 6 ) ;

	return( bt ) ;
}

void read_DWI_file( float *S, char *sr )	// read raw file in vector S
{
	long l ;
	unsigned char src[4] ;
	FILE *f ;

	if( ( f = fopen( sr , "rb" ) ) == NULL ) {
		printf ( "Cannot open input file.\n" ) ;
		exit ( 1 ) ;
	}

	for( l = 0 ; l < (N+1)*M1*M2*M3 ; l ++ ) {
		fread( src, sizeof( unsigned char ), 4, f ) ;
		S[l] = swap( src ) ;
	}
```

```
        fclose( f ) ;
}

void calculate_DTI( float *DTI, float *S, float **btbibt, float S_thr, float D_thr )
{
        int i,j,k,nrot ;
        long l ;
        float I[N],D6[6],**D,**e,*Dl ;
        float S0,sum,MD,FA ;

        D = matrix( 1, 3, 1, 3 ) ;
        e = matrix( 1, 3, 1, 3 ) ;
        Dl = vector( 1, 3 ) ;

        for( l = 0 ; l < M1*M2*M3 ; l++ ) {
                S0 = S[l] ;
                if( S0 > S_thr ) {

                        for( k = 0 ; k < N ; k++ ) I[k] = -logf(S[(k+1)*M1*M2*M3+l]/S0) ;

                        for( i = 0 ; i < 6 ; i++ ) {        // D in the laboratory system
                                sum = 0.0 ;
                                for( k = 0 ; k < N ; k++ ) sum += btbibt[i+1][k+1]*I[k] ;
                                D6[i] = 1000.0 * sum ;       // in 10-9 m2/s
                        }

                        D[1][1] = D6[0] ;
                        D[2][2] = D6[1] ;
                        D[3][3] = D6[2] ;
                        D[1][2] = D[2][1] = D6[3] ;
                        D[1][3] = D[3][1] = D6[4] ;
                        D[2][3] = D[3][2] = D6[5] ;

                        jacobi( D, 3, Dl, e, &nrot ) ;      // solving eigenvalues and
                                                            // eigenvectors

                        eigsrt( Dl, e, 3 ) ;                // to sort eigenvalues

                        // eigenvalues, ordered

                        if( (Dl[1] >= D_thr ) && (Dl[2] >= 0.0) && (Dl[3] >= 0.0) ) {

                                // mean diffusivity

                                MD = (Dl[1] + Dl[2] + Dl[3]) / 3.0 ;

                                // fractional anisotorpy

                                FA = sqrtf(3.0*(sqr(Dl[1]-MD)+sqr(Dl[2]-MD)+sqr(Dl[3]-MD))) ;
                                FA /= sqrtf(2.0*(sqr(Dl[1])+sqr(Dl[2])+sqr(Dl[3]))) ;

                                // eigenvectors e[i][j] : i-x,y,z components, j-vectors

                                DTI[L*l] = Dl[1] ;
                                DTI[L*l+1] = Dl[2] ;
                                DTI[L*l+2] = Dl[3] ;
                                DTI[L*l+3] = MD ;
                                DTI[L*l+4] = FA ;

                        }
                        else for( i = 0 ; i < L ; i++ ) DTI[L*l+i] = 0.0 ;
                }
                else for( i = 0 ; i < L ; i++ ) DTI[L*l+i] = 0.0 ;
        }
```

```
        free_matrix( D, 1, 3, 1, 3 ) ;
        free_matrix( e, 1, 3, 1, 3 ) ;
        free_vector( Dl, 1, 3 ) ;
}

void write_DTI_file( float *DTI )    // write maps of eigenvalues, MD and FA
{
        // DTI file contains a sequence of five maps:
        // Dl1, Dl2, Dl3, MD, FA

        int i ;
        long l ;
        FILE *f ;

        if( ( f = fopen( "DTI.raw" , "wb" ) ) == NULL ) {
                printf( "Cannot open file \n" ) ;
                exit( 1 ) ;
        }

        for( i = 0 ; i < L ; i ++ ) {
                for( l = 0 ; l < M1*M2*M3 ; l ++ ) {
                        fwrite( &DTI[L*l+i] , sizeof(float) , 1 , f ) ;
                }
        }

        fclose( f ) ;
}

float calculate_CN( float **btbibt )        // calculate condition number
{
        int i,j ;
        float **A,*w,**V ;

        A = matrix( 1, N, 1, N ) ;
        w = vector( 1, N ) ;
        V = matrix( 1, N, 1, N ) ;

        for( i = 1 ; i <= N ; i++ ) {
                for( j = 1 ; j <= N ; j++ ) {
                        if( i <= 6 ) A[i][j] = btbibt[i][j] ;
                        else A[i][j] = 0.0 ;
                }
        }

        svdcmp( A, N, N, w, V ) ;
        for( i = 1 ; i <= N ; i++ ) w[i] = fabs( w[i] ) ;
        piksrt( N, w ) ;

        free_matrix( A, 1, N, 1, N ) ;
        free_vector( w, 1, N ) ;
        free_matrix( V, 1, N, 1, N ) ;

        return( w[N]/w[N-5] ) ;
}

int main( int argc, char *argv[] )   // main procedure
{
        float **btbibt,*S ;
        float b0,S_thr,D_thr ;
        float *DTI ;

        // parameters are inserted in the command line

        if( argc < 5 ) {
                printf( "Command format: DTI DWI-raw_image N b threshold\n" ) ;
                printf( "                DWI-raw_image – DW image raw file\n" ) ;
```

```
        printf( "                N - gradient orientations (6 or 19)\n" ) ;
        printf( "                b - b-value in s/mm2\n" ) ;
        printf( "                S_thr - signal threshold of the b=0 image\n" ) ;
        printf( "                D_thr - diffusion threshod in 10-9 m2/s\n" ) ;

        exit( 0 ) ;
    }
    sscanf( argv[2] , "%d" , &N ) ;
    sscanf( argv[3] , "%f" , &b0 ) ;
    sscanf( argv[4] , "%f" , &S_thr ) ;
    sscanf( argv[5] , "%f" , &D_thr ) ;

    S = vector( 0 , (N+1)*M1*M2*M3-1 ) ;
    DTI = vector( 0, L*M1*M2*M3-1 ) ;

    btbibt = define_btbibt( b0 ) ;
    read_DWI_file( S, argv[1] ) ;
    calculate_DTI( DTI, S, btbibt, S_thr, D_thr ) ;
    write_DTI_file( DTI ) ;
    printf( "Condition number = %f\n" , calculate_CN( btbibt ) ) ;

    free_vector( S, 0, (N+1)*M1*M2*M3-1 ) ;
    free_vector( DTI, 0, L*M1*M2*M3-1 ) ;
    free_matrix( btbibt, 1, 6, 1, N ) ;

    return( 0 ) ;
}
```

```
#include <stdio.h>                  // program DTI_3D_ellips.c
#include <stdlib.h>
#include <math.h>

#include "../../recipes/nrutil.c"   // libraries of Numerical recipes in C
#include "../../recipes/gaussj.c"
#include "../../recipes/jacobi.c"
#include "../../recipes/eigsrt.c"
#include "../../recipes/svdcmp.c"
#include "../../recipes/piksrt.c"

#define M1 40         // image matrix size is M1 * M2 * M3 (x y z)
#define M2 40
#define M3 12
int N ;               // number of different gradient orientations
#define L 9           // images (data) per pixel
#define D_max 1.6     // 1.6 10-9 m2/s is the diffusion range for display
#define dx 1.0        // voxel size x-direction
#define dy 1.0        // voxel size y-direction
#define dz 4.44       // voxel size z-direction
#define eps 0.0001    // tolerance for angle calculation

#define sqr(x) ( (x) * (x) )
#define mag( x , y ) sqrtf( (x) * (x) + (y) * (y) )
#define deg(x) ( (int)(57.2958*(x) + 0.5) )

const float g6[6][3] = { {  0.0000,  0.7071,  0.7071 },   // diffusion gradient directions
                         {  0.0000, -0.7071,  0.7071 },
                         { -0.7071,  0.0000,  0.7071 },
                         { -0.7071,  0.0000, -0.7071 },
                         { -0.7071,  0.7071,  0.0000 },
                         { -0.7071, -0.7071,  0.0000 } } ;

const float g19[19][3] = { { -0.0620,  0.2380,  0.9693 }, // diffusion gradient directions
                           {  0.6092,  0.7125,  0.3483 },
                           { -0.7878,  0.3181, -0.5274 },
                           {  0.0636,  0.1273,  0.9898 },
                           { -0.8853, -0.0582,  0.4613 },
                           { -0.1428, -0.8146,  0.5622 },
                           {  0.4337, -0.8094, -0.3959 },
                           {  0.2289, -0.4735,  0.8505 },
                           { -0.6445, -0.2860, -0.7091 },
                           { -0.4461,  0.3051,  0.8414 },
                           {  0.0199, -0.9966, -0.0799 },
                           { -0.7535, -0.5805,  0.3086 },
                           { -0.8716,  0.4899, -0.0185 },
                           { -0.4636,  0.7949, -0.3914 },
                           { -0.7682,  0.4252,  0.4787 },
                           { -0.1220, -0.6652, -0.7367 },
                           { -0.9632, -0.1645, -0.2125 },
                           {  0.3718,  0.3251, -0.8696 },
                           { -0.4298, -0.8959,  0.1125 } } ;

float swap(unsigned char *src)      // convert big-endian to little-endian
{
       float a;
       unsigned char *dst = (unsigned char *)&a;

       dst[0] = src[3];
       dst[1] = src[2];
       dst[2] = src[1];
       dst[3] = src[0];

       return a;
}
```

```
float** define_btbibt( float b0 )   // matrix product on righthand side of Eq. 14
{
       float **b,**bt,**btb ;
       float sum ;
       int i,j,k ;

       b = matrix( 1, N, 1, 6 ) ;
       bt = matrix( 1, 6, 1, N ) ;
       btb = matrix( 1, 6, 1, 6 ) ;

       switch( N ) {
              case 6 :
              for( i = 0 ; i < N ; i++ ) {
                     b[i+1][1] = b0 * g6[i][0] * g6[i][0] ;
                     b[i+1][2] = b0 * g6[i][1] * g6[i][1] ;
                     b[i+1][3] = b0 * g6[i][2] * g6[i][2] ;
                     b[i+1][4] = b0 * 2.0 * g6[i][0] * g6[i][1] ;
                     b[i+1][5] = b0 * 2.0 * g6[i][0] * g6[i][2] ;
                     b[i+1][6] = b0 * 2.0 * g6[i][1] * g6[i][2] ;
              } break ;
              case 19 :
              for( i = 0 ; i < N ; i++ ) {
                     b[i+1][1] = b0 * g19[i][0] * g19[i][0] ;
                     b[i+1][2] = b0 * g19[i][1] * g19[i][1] ;
                     b[i+1][3] = b0 * g19[i][2] * g19[i][2] ;
                     b[i+1][4] = b0 * 2.0 * g19[i][0] * g19[i][1] ;
                     b[i+1][5] = b0 * 2.0 * g19[i][0] * g19[i][2] ;
                     b[i+1][6] = b0 * 2.0 * g19[i][1] * g19[i][2] ;
              } break ;
              default :
                     printf( "No such gradient table!\n" ) ;
                     exit( 1 ) ;
       }

       for( i = 1 ; i <= N ; i++ ) {
              for( j = 1 ; j <= 6 ; j++ ) {
                     bt[j][i] = b[i][j] ;
              }
       }

       for( i = 1 ; i <= 6 ; i++ ) {
              for( j = 1 ; j <= 6 ; j++ ) {
                     sum = 0.0 ;
                     for( k = 1 ; k <= N ; k++ ) sum += bt[i][k] * b[k][j] ;
                     btb[i][j] = sum ;
              }
       }

       gaussj( btb, 6, bt, N ) ;    // Gauss-Jordan elimination to compute (btb)-1bt
                                    // the result is stored in bt, btb holds its inverse
       free_matrix( b, 1, N, 1, 6 ) ;
       free_matrix( btb, 1, 6, 1, 6 ) ;

       return( bt ) ;
}

void read_DWI_file( float *S, char *sr )    // read raw file in vector S
{
       long l ;
       unsigned char src[4] ;
       FILE *f ;

       if( ( f = fopen( sr , "rb" ) ) == NULL ) {
              printf ( "Cannot open input file.\n" ) ;
              exit ( 1 ) ;
       }
```

```
	for( l = 0 ; l < (N+1)*M1*M2*M3 ; l ++ ) {
		fread( src, sizeof( unsigned char ), 4, f ) ;
		S[l] = swap( src ) ;
	}

	fclose( f ) ;
}

void calc_angles( float **e, float *alpha, float *beta, float *gamma )
{
	int i ;                               // routine to convert eigenvectors to
	float al,be,gm,al0,v2x,v2y,cnd ;      // direction angles of the main eigenvector

	if(fabs(e[3][2]/e[3][3]) < 1) al0 = atan( e[3][2]/e[3][3] ) ;
	else al0 = M_PI_2 - atan( e[3][3]/e[3][2] ) ;
	be = - asin( e[3][1] ) ;
	if(fabs(e[2][1]/e[1][1]) < 1) gm = - atan( e[2][1]/e[1][1] ) ;
	else gm = M_PI_2 + atan( e[1][1]/e[2][1] ) ;

	if(fabs(cos(be)*cos(gm) - e[1][1]) > eps) be = M_PI - be ;
	i = 0 ;
	do {
		i++ ;
		switch( i ) {
		case 1 : al = al0 ; break ;
		case 2 : al = M_PI - al0 ; break ;
		case 3 : al = -al0 ; break ;
		case 4 : al = -M_PI + al0 ; break ;
		}
		v2x = sin(al)*sin(be)*cos(gm) + cos(al)*sin(gm) ;
		v2y = -sin(al)*sin(be)*sin(gm) + cos(al)*cos(gm) ;
		cnd = fabs(v2x - e[1][2]) + fabs(v2y - e[2][2]) ;
	} while((cnd > eps) && (i<4)) ;

	if(al < 0) al += 2*M_PI ;
	if(be < 0) be += 2*M_PI ;
	if(gm < 0) gm += 2*M_PI ;

	*alpha = al ;
	*beta = be ;
	*gamma = gm ;
}

void calculate_DTI( float *DTI, float *S, float **btbibt, float S_thr, float D_thr )
{
	int i,j,k,nrot ;
	long l ;
	float I[N],D6[6],**D,**e,*Dl ;
	float S0,sum,alpha,beta,gamma ;

	D = matrix( 1, 3, 1, 3 ) ;
	e = matrix( 1, 3, 1, 3 ) ;
	Dl = vector( 1, 3 ) ;

	for( l = 0 ; l < M1*M2*M3 ; l++ ) {
		S0 = S[l] ;
		if( S0 > S_thr ) {

			for( k = 0 ; k < N ; k++ ) I[k] = -logf(S[(k+1)*M1*M2*M3+l]/S0) ;

			for( i = 0 ; i < 6 ; i++ ) {         // D in the laboratory system
				sum = 0.0 ;
				for( k = 0 ; k < N ; k++ ) sum += btbibt[i+1][k+1]*I[k] ;
				D6[i] = 1000.0 * sum         // in 10-9 m2/s
			}
```

```
                    D[1][1] = D6[0] ;
                    D[2][2] = D6[1] ;
                    D[3][3] = D6[2] ;
                    D[1][2] = D[2][1] = D6[3] ;
                    D[1][3] = D[3][1] = D6[4] ;
                    D[2][3] = D[3][2] = D6[5] ;

                    jacobi( D, 3, Dl, e, &nrot ) ;       // solving eigenvalues and
                                                         // eigenvectors

                    eigsrt( Dl, e, 3 ) ;                 // to sort eigenvalues

                    // eigenvalues, ordered

                    if( (Dl[1] >= D_thr ) && (Dl[2] >= 0.0) && (Dl[3] >= 0.0) ) {

                           calc_angles( e, &alpha, &beta, &gamma ) ;

                           // eigenvectors e[i][j] : i-x,y,z components, j-vectors

                           DTI[L*l] = Dl[1] ;
                           DTI[L*l+1] = Dl[2] ;
                           DTI[L*l+2] = Dl[3] ;
                           DTI[L*l+3] = e[1][1] ;
                           DTI[L*l+4] = e[2][1] ;
                           DTI[L*l+5] = e[3][1] ;
                           DTI[L*l+6] = alpha ;
                           DTI[L*l+7] = beta ;
                           DTI[L*l+8] = gamma ;

                    }
                    else for( i = 0 ; i < L ; i++ ) DTI[L*l+i] = 0.0 ;
             }
             else for( i = 0 ; i < L ; i++ ) DTI[L*l+i] = 0.0 ;
       }

       free_matrix( D, 1, 3, 1, 3 ) ;
       free_matrix( e, 1, 3, 1, 3 ) ;
       free_vector( Dl, 1, 3 ) ;
}

void write_DTI_file( float *DTI )   // write POV-Ray commands for displaying ellipsoids
{
       // DTI vector contains a sequence of five maps:
       // Dl1, Dl2, Dl3, e[1][1], e[2][1], e[3][1], alpha, beta, gamma

       int i,j,k ;
       long l ;
       float nf,MD,FA ;
       FILE *f ;

       if( ( f = fopen( "DTI_3D_ellips.txt" , "wt" ) ) == NULL ) {
             printf( "Cannot open file \n" ) ;
             exit( 1 ) ;
       }

       l = 0 ;
       nf = 1.0 / D_max ;
       for( k = 0 ; k < M3 ; k+=2 ) {
             for( j = 0 ; j < M2 ; j++ ) {
                    for( i = 0 ; i < M1 ; i++ ) {
                           if( (DTI[L*l] > 0) && (DTI[L*l] < D_max) ) {
                                  MD = (DTI[L*l] + DTI[L*l+1] + DTI[L*l+2]) / 3.0 ;
                                  FA = sqrtf( 3.0*(sqr(DTI[L*l]-MD) + sqr(DTI[L*l+1]-MD)
                                       + sqr(DTI[L*l+2]-MD)) ) ;
```

```
                                    FA /= sqrtf( 2.0*(sqr(DTI[L*l]) + sqr(DTI[L*l+1])
                                          + sqr(DTI[L*l+2])) ) ;

                                    if( (FA > 0.1) && ( FA < 0.7) ) {

fprintf( f, "sphere {<0,0,0>,0.5 scale<%4.2f,%4.2f,%4.2f> ", nf*DTI[L*l], nf*DTI[L*l+1],
nf*DTI[L*l+2] ) ;
fprintf( f, "rotate<%d,%d,%d> ", deg(DTI[L*l+6]), deg(DTI[L*l+7]), deg(DTI[L*l+8]) ) ;
fprintf( f, "translate<%4.2f,%4.2f,%4.2f> ", dx*(i-M1/2), dy*(M2/2-j-1), -dz*(M3/2-k-1) ) ;
fprintf( f, "pigment{color rgb<%4.2f,%4.2f,%4.2f>}}\n", fabs(DTI[L*l+3]), fabs(DTI[L*l+4]),
fabs(DTI[L*l+5]) ) ;
                                    }
                              }
                              l ++ ;
                        }
                  }
                  l += M1*M2 ;
      }

      fclose( f ) ;
}

int main( int argc, char *argv[] )  // main procedure
{
      float **btbibt,*S ;
      float b0,S_thr,D_thr ;
      float *DTI ;

      if( argc < 5 ) {
            printf( "Command format: DTI DWI-raw_image N b threshold\n" ) ;
            printf( "                DWI-raw_image – DW image raw file\n" ) ;
            printf( "                N - gradient orientations (6 or 19)\n" ) ;
            printf( "                b - b-value in s/mm2\n" ) ;
            printf( "                S_thr - signal threshold of the b=0 image\n" ) ;
            printf( "                D_thr - diffusion threshod in 10-9 m2/s\n" ) ;

            exit( 0 ) ;
      }
      sscanf( argv[2] , "%d" , &N ) ;
      sscanf( argv[3] , "%f" , &b0 ) ;
      sscanf( argv[4] , "%f" , &S_thr ) ;
      sscanf( argv[5] , "%f" , &D_thr ) ;

      S = vector( 0 , (N+1)*M1*M2*M3-1 ) ;
      DTI = vector( 0, L*M1*M2*M3-1 ) ;

      btbibt = define_btbibt( b0 ) ;
      read_DWI_file( S, argv[1] ) ;
      calculate_DTI( DTI, S, btbibt, S_thr, D_thr ) ;
      write_DTI_file( DTI ) ;

      free_vector( S, 0, (N+1)*M1*M2*M3-1 ) ;
      free_vector( DTI, 0, L*M1*M2*M3-1 ) ;
      free_matrix( btbibt, 1, 6, 1, N ) ;

      return( 0 ) ;
}
```

```
#version 3.7;                              // nerve_ellips.pov
global_settings {assumed_gamma 1.0}        // POV-Ray program for displaying ellipsoids

#include "colors.inc"

camera {
        //perspective //keyword is facultative in this case
        orthographic
        location <600,400,200>
        //right    x*image_width/image_height
        look_at  <0,0,4>
        angle    4.5
       }

background { White }
light_source {<300,200,100> White*1.5}

#declare base_vectors = merge {
   cylinder { <0,0,0>,<10,0,0>,0.5 pigment{Red} }
   cone { <10,0,0>,1,<12,0,0>,0 pigment{Red} }
   cylinder { <0,0,0>,<0,10,0>,0.5 pigment{Green} }
   cone { <0,10,0>,1,<0,12,0>,0 pigment{Green} }
   cylinder { <0,0,0>,<0,0,10>,0.5 pigment{Blue} }
   cone { <0,0,10>,1,<0,0,12>,0 pigment{Blue} }
}

object{ base_vectors scale<0.4,0.4,0.4> translate<10,-10,-18> }

#include "DTI_3D_ellips.txt"
```
